# An Efficient Ionic Liquid-Mediated Extraction and Enrichment of Isoimperatorin from *Ostericum koreanum* (Max.) Kitagawa

**DOI:** 10.3390/molecules26216555

**Published:** 2021-10-29

**Authors:** Alice Nguvoko Kiyonga, Gyu Hwan Park, Hyun Su Kim, Young-Ger Suh, Tae Kon Kim, Kiwon Jung

**Affiliations:** 1 Institute of Pharmaceutical Sciences, College of Pharmacy, CHA University, Sungnam 13844, Korea; gabriella@chauniv.ac.kr (A.N.K.); khs8812@snu.ac.kr (H.S.K.); ygsuh@cha.ac.kr (Y.-G.S.); 2 Research Institute of Pharmaceutical Sciences, College of Pharmacy, Kyungpook National University, Daegu 41566, Korea; park014@knu.ac.kr; 3 College of Science and Engineering, Jungwon University, Geosan-gun, Chungbuk 28024, Korea

**Keywords:** ionic liquids, isoimperatorin, extraction, crystallization, enrichment, *Ostericum koreanum*

## Abstract

Ionic liquids (ILs) have attracted significant interest because of their desirable properties. These characteristics have improved their application to overcome the shortcomings of conventional separation techniques for phytochemicals. In this study, several ILs were investigated for their capacity to extract isoimperatorin, a bioactive furanocoumarin, from the roots of *Ostericum koreanum*. Herein, 1-Butyl-3-methylimidazolium tetrafluoroborate ([Bmim][BF_4_]) was selected as a promising IL for separating isoimperatorin. A central composite design was applied to optimize the extraction conditions. Under the optimal conditions, the yield of isoimperatorin reached 97.17 ± 1.84%. Additionally, the recovery of isoimperatorin from the [Bmim][BF_4_] solution was successfully achieved (87.73 ± 2.37%) by crystallization using water as an antisolvent. The purity of the isoimperatorin was greatly enhanced, from 0.26 ± 0.28% in the raw material to 26.94 ± 1.26% in the product, in a one-step crystallization process. Namely, an enhancement of approximately 103-folds was reached. The developed approach overcomes the shortcomings of conventional separation methods applied for gaining isoimperatorin by significantly reducing the laboriousness of the process and the consumption of volatile organic solvents. Moreover, the simplicity and effectiveness of the method are assumed to be valuable for producing isoimperatorin-enriched products and for promoting its purification. This work also confirms the efficiency of ILs as a promising material for the separation of phytochemicals.

## 1. Introduction

Ionic liquids (ILs) refer to salts that are principally composed of organic cations paired with organic or inorganic anions, and whose melting point is below 100 °C [1]. ILs have been applied in different fields and are considered to be potential substitutes for volatile organic solvents, owing to their exceptional characteristics [2]. These include great selectivity, negligible vapor pressure, inflammability, high thermal stability, tunability, and the strong ability to dissolve a large variety of compounds [3,4,5,6,7,8]. In analytical chemistry, studies have demonstrated that ILs are promising solvents for the separation of diverse phytochemicals, including phenolics [9], alkaloids [10,11], flavonoids [12,13], ginsenosides [14], terpenoids [15,16], coumarins [17] and so on [18,19].

The recovery of bioactive components from IL solutions after their extraction remains a crucial challenge. Nevertheless, some techniques, like back extraction [17], supercritical fluid extraction [20], microporous resin [21], and crystallization [22,23], have been successfully implemented for recovering plant bioactive components from IL solutions. However, more studies are still urgently required to improve the application of ILs. Crystallization is a method by which a solution, a liquid, or a gas undergoes a physical transformation to produce a highly organized solid form called crystal. Crystallization is preferred by scientists because of its simplicity, selectivity, and efficiency in producing high purity compounds [24]. Kiyonga et al. [25] developed an effective crystallization method for recovering decursin and decursinol angelate from the [BMIm][BF_4_] IL extraction solution of the medicinal plant, *Angelica gigas* Nakai. Two compounds were gained with great purity and yields.

**Figure 1:**
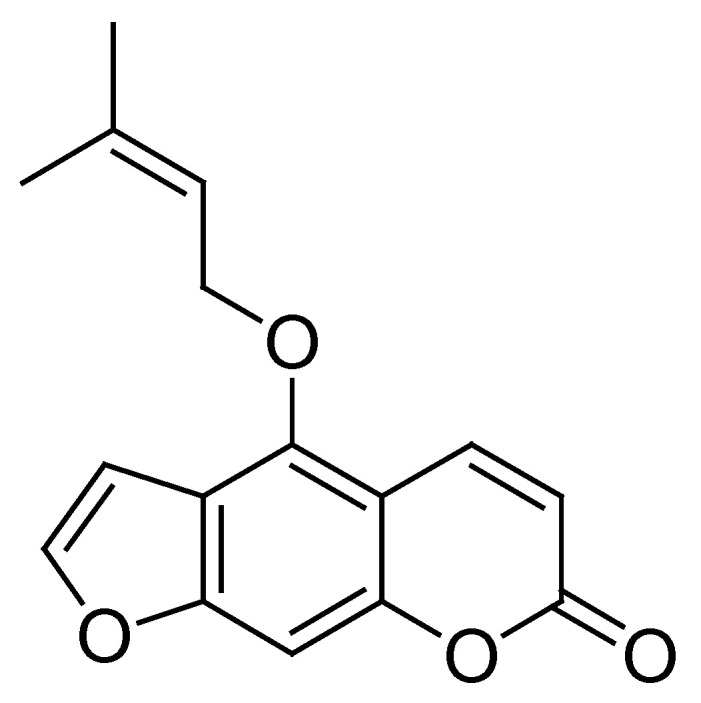
Chemical structure of isoimperatorin.

*Ostericum koreanum* (Max.) Kitagawa (*O. koreanum*) is an important Korean medicinal herb which has been used to prevent and treat cold and headache symptoms, neuralgia, and arthritis [26]. Active components responsible for the pharmacological properties include essential oils, isoimperatorin, oxypeucedanin, isooxypeucedanin, bergapten, osthol, oxypeucedanin hydrate, cnidicin, marmesinin, chromones, bisabolangelone, etc. [27,28,29,30,31]. Isoimperatorin (Figure 1) is a naturally occurring furanocoumarin present in this herb, and it has been reported that isoimperatorin exhibits potent antitumor [32,33,34], antibacterial [35,36], anti-inflammatory [37], analgesic [38], antioxidant [39,40,41] and antiviral [42,43] properties. Most studies involving the separation of isoimperatorin and related compounds from the roots of *O. koreanum* focus on the determination of relative quantities and biological efficacies, as well as the origin discrimination of the plant materials [44,45,46]. In addition, the separation processes of isoimperatorin and related compounds reported in these works are laborious and demand multiple chromatographic processes [45,46]. To the best of our knowledge, a study solely focusing on the efficient separation and/or enrichment of isoimperatorin from the roots of *O. koreanum* has not been reported to date. Therefore, the present work aimed to develop a straightforward process for the extraction, recovery, and enrichment of isoimperatorin from the roots of *O.* *koreanum*. ILs were used as extractants and a crystallization technique was applied for the recovery and enrichment of the targeting component from the IL solutions. Several variables were rigorously investigated and statistically optimized using response surface methodology (RSM). The ultimate purpose was to assess the potentiality of ILs as separation solvent for phytochemicals.

## 2. Results and Discussion

### 2.1. Selection of IL

**Figure 2:**
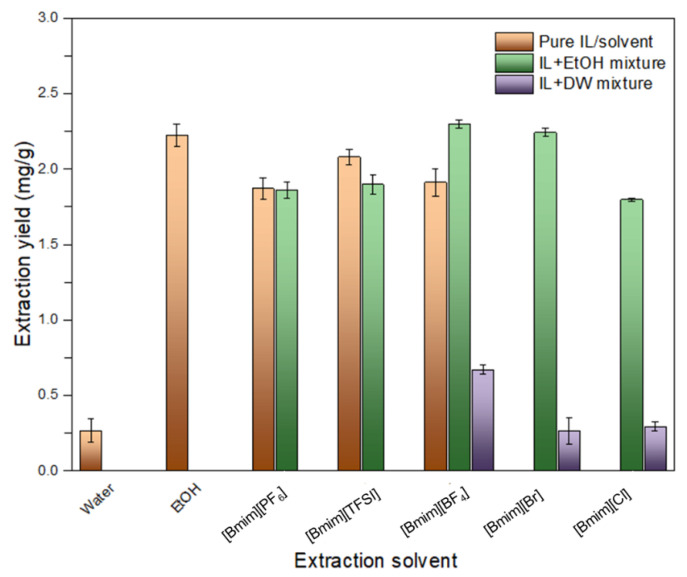
Effect of extraction media on the extraction yield of isoimperatorin.

Experimental processes were conducted using the following Imidazolium-based ILs: 1-Butyl-3-methylimidazolium hexafluorophosphate ([BMIm][PF_6_]), 1-Butyl-3-methylimidazolium (trifluoromethylsulfonyl)imide ([BMIm][TFSI]), 1-Butyl-3-methylimidazolium bromide ([Bmim][Br]), 1-Butyl-3-methylimidazolium tetrafluoroborate ([Bmim][BF_4_]) and 1-Butyl-3-methylimidazolium chloride ([Bmim][Cl]). The aim was to investigate the effects of IL-cosolvent mixtures and pure ILs (case of room temperature ILs) in improving the extraction yield of isoimperatorin. Ethanol (EtOH) and deionized water (water) were used as cosolvents. IL-cosolvent mixtures have been used in several studies for the extraction of various bioactive compounds. IL-cosolvent systems are used for their abilities to reduce the viscosity of ILs and to tune the solvent properties [47]. Herein, samples were extracted on a hot plate for 30 min and then sonicated at 40 kHz frequency for 30 min. The content of IL in the cosolvent system was fixed as one mole per liter (1.0 mol/L) and the solid to liquid ratio was fixed at 1:10. Supplementary experiments were conducted under identical experimental conditions using 98% EtOH and water to compare the extraction ability of these commonly used solvents with the IL systems. As shown in Figure 2, [Bmim][BF_4_]-EtOH, [Bmim][Br]-EtOH and 98% EtOH solutions demonstrated the greatest extraction yields for isoimperatorin. However, preliminary crystallization experiments were carried out on all extracts to select the IL that simultaneously possesses acceptable extraction and recovery performances for isoimperatorin. Aqueous IL extracts were excluded because of their lower extraction ability for isoimperatorin. Methanol (MeOH), EtOH and water were used as antisolvents. From the preliminary recovery tests (results not shown), precipitates were solely observed from pure [Bmim][BF_4_] IL, 98% EtOH, [Bmim][Br]-EtOH and [Bmim][BF_4_]-EtOH solutions when water was utilized as antisolvent, and the rate of recovery of isoimperatorin was satisfying. Nonetheless, it was observed that precipitate yielded from pure [Bmim][BF_4_] solution contained a greatly reduced number of unwanted components compared to others. The lower aqueous solubility of isoimperatorin, namely, its supersaturation level is presumed as the driving force that led to the precipitation of isoimperatorin. It was also suggested that the change in both recovery and purity of isoimperatorin in the products has been impacted by the nature, the properties of the crystallization system (solvent/antisolvent mixture), and the system viscosity. Based on the above, pure [Bmim][BF_4_] was selected as an adequate IL for further experiments.

### 2.2. Optimization of the Variables

The univariate and multivariate statistical analyses were used to optimize the extraction method. The extraction medium was [Bmim][BF_4_]. The following were the variables investigated: sonication time, extraction time, solid to liquid ratio, and extraction temperature.

#### 2.2.1. Effect of Sonication Time

**Figure 3:**
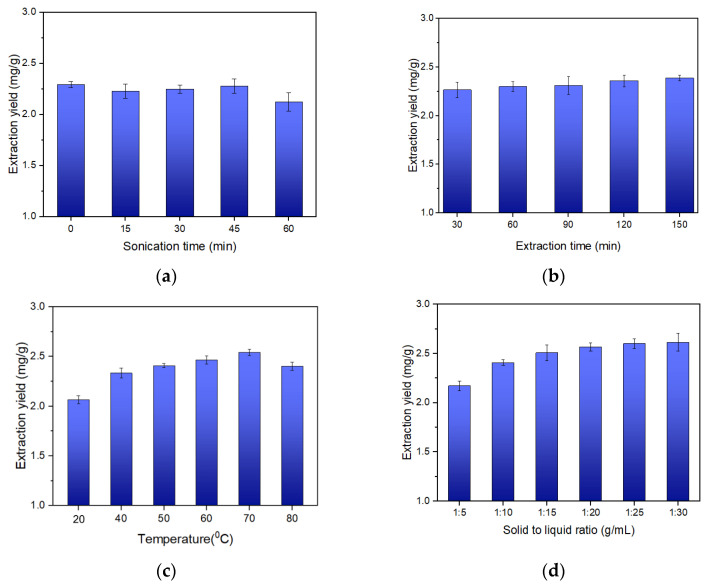
Effect of several variables on the extraction efficiency of isoimperatorin. (**a**) effect of sonication time, (**b**) effect of extraction time, (**c**) effect of solid to liquid ratio, (**d**) effect of solid to liquid ratio.

Ultrasonic-assisted extraction is known to increase the extraction yield of phytochemicals. This is because it facilitates solvent penetration into the sample matrix and favors contact between the solvent and the solid material [48,49]. The samples were extracted on a hot plate for 30 min and then sonicated at various times; 0 min, 15 min, 30 min, 45 min, and 60 min. Longer sonication times were avoided to prevent the decomposition of compounds after prolonged exposure to ultrasonic irradiation. The results displayed in Figure 3a show that the extracts obtained without sonication possessed similar extraction yields to those obtained after sonication (up to 45 min), indicating the time of sonication exerted a negligible effect on the extraction yield of isoimperatorin at the experimental ultrasound frequency. However, a slight decrease in the extraction yield was observed at 60 min. The overall result can be explained by the acoustic cavitation phenomenon. During ultrasonic extraction, cavitation bubbles are formed with regard to ultrasound power, and later collapse with greater energy if the acoustic pressure is sufficient. The collapse of bubbles near the surface of solid matter generates fragmentation of the matrix, which consequently facilitates solvent penetration and extraction [50,51]. However, the acoustic cavitation phenomenon is influenced by the solvent viscosity [50,51,52]. High viscosity reduces the intensity of bubble formation, hindering the rapid solvent penetration in the matrix and, thus, the extraction. In this regard, the relatively high viscosity of the [Bmim][BF_4_] IL is assumed to have affected the performance of the experimental sonication power and time. This led to negligible improvement in the extraction yield of isoimperatorin. Considering the above, sonication was excluded in the following experiments.

#### 2.2.2. Effect of Extraction Time

In the aim of improving the extraction yield of isoimperatorin, the extraction process was carried out under various extraction time variables; 30 min, 60 min, 90 min, 120 min, and 150 min. From the results in Figure 3b, it can be seen that there was no apparent change in the extraction yield of isoimperatorin within the experimental time range. However, 90 min was arbitrarily selected for the following experiments in order to prevent uncompleted extraction.

#### 2.2.3. Effect of Temperature

The temperature variable was studied to evaluate its impact on the extraction yield of isoimperatorin. The temperatures were varied from 20 to 80 °C. As seen in Figure 3c, the extraction yield of isoimperatorin slightly increased in the range between 20 to 70 °C. A negligible decrement in the extraction yield was observed at 80 °C. Chen et al. [53] observed that isoimperatorin was relatively stable in the water/ethanol mixture within the experimental temperature range from 20 to 80 °C. However, in the present study, isoimperatorin was found to be stable up to 70 °C, when using [Bmim][BF_4_] IL; indicating that isoimperatorin undergoes degradation at elevated temperatures. In addition, it was also noted that the content of unwanted compounds increased at elevated temperatures. This compelled us to conduct preliminary recovery experiments to examine the impact of the higher content of unwanted compounds on the recovery yield and the purity of isoimperatorin in the products. From the findings, 70 °C demonstrated a slightly high recovery yield of isoimperatorin compared to 50 and 60 °C. However, products with highly improved purity of isoimperatorin were acquired from the 50 °C extracts. This is because the unwanted components which increased at elevated temperatures tended to precipitate during the crystallization process. The increase in the content of unwanted compounds led to a significant decrement of isoimperatorin purity in the recovered products. Because 50 °C showed satisfactory recovery and the highest purity of isoimperatorin when compared with 60 °C and 70 °C, it was selected as an adequate extraction temperature for further experiments. At 50 °C the extraction yield of isoimperatorin was 2.41 mg/g (91.57%).

#### 2.2.4. Effect of Solid to Liquid Ratio

Experiments were performed to investigate the impact of the solid to liquid ratio (1:5, 1:10, 1:15, 1:20, 1:25, and 1:30) on the extraction efficiency of isoimperatorin. The purpose was to improve the extraction yield of isoimperatorin, preventing uncompleted extraction, and avoiding waste of the solvent. As a result, the extraction yield of isoimperatorin increased from 2.17 mg/g (82.59%) to 2.57 mg/g (97.63%) when the solid to liquid ratio was increased from 1:5 to 1:20. A slight improvement was observed at higher ratios (Figure 3d). The result can be explained by the fact that a larger volume of the solvent enhances the mass transfer, increases the dissolution rate of the compound of interest, and therefore improves its extraction yield.

### 2.3. Statistical Analysis

A central composite design (CCD) was applied in order to investigate the correlation between the extraction time (X_1_) and solid to liquid ratio (X_2_) variables on the extraction yield of isoimperatorin. The critical variables and experimental runs illustrated in Table 1 were established based on the data derived in Section 2.2. Variables were examined at five levels (−α, −1, 0, +1, +α) that were predicted by CCD. The extraction temperature and rotational speed were fixed at 50 °C and 600 rpm, respectively. The experimental results are illustrated in Table 1.

Table 2 illustrates the coefficients for the fitted models as well as the validity of the models based on the regression coefficients, R^2^ and R^2^ -adj, and the lack of fit of P and F-values. The X_2_, X_1_^2^ and X_2_^2^ coefficients were significant with P-values inferior to 0.05. This indicates that the solid to liquid ratio has a huge impact on the extraction of isoimperatorin. The result also suggests that the longer the extraction time, the greater the extraction yield of isoimperatorin. In addition, the values of R^2^ (98.34%) and R^2^-adj (97.16%), fit of *P*-values (*p* = 0.132) and *F*-values (*F* = 3.44) confirm that the generated models are suitable to predict the responses. The relationship between the responses and the variables is expressed by the equation.

Yield of isoimperatorin = 93.0555 + 2.2010X_1_ + 22.3526X_2_ − 4.5834X_1_^2^ − 12.7052 X_2_^2^ + 0.2934X_1_X_2_ (1)

**Figure 4:**
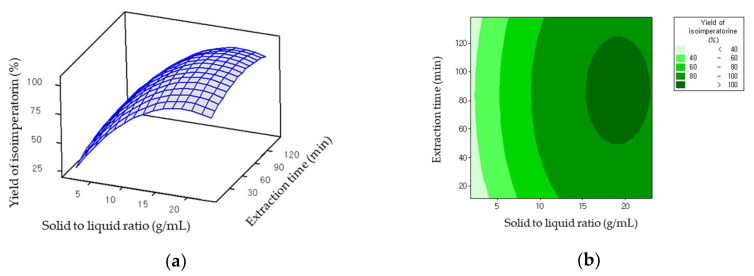
Plots of the extraction yield of isoimperatorin versus variables. (**a**) Three-dimensional response surface plot, (**b**) Contour plot.

The three-dimensional response surface and contour plots in Figure 4a,b show the effect of the interaction between the extraction time and solid to liquid ratio variables. The optimum conditions proposed by CCD were 46 min extraction time and 1:19 solid to liquid ratio to produce a maximum yield of 98.85% (2.59 mg/g) isoimperatorin. The temperature and rotational speed were fixed at 50 °C and 600 rpm, respectively. Under the optimum experimental conditions, conducted in triplicate, the extraction yield of isoimperatorin was 97.17 ± 1.89% (2.56 ± 0.05 mg/g). From the confirmation experiments, it was observed that the predicted and experimental response values were extremely close. Accordingly, the RSM model is proved suitable to estimate the extraction yield of isoimperatorin from the roots of *O. Koreanum* by using [Bmim][BF_4_].

### 2.4. Recovery of Isoimperatorin from the [Bmim][BF_4_] IL Extraction Solution

**Figure 5:**
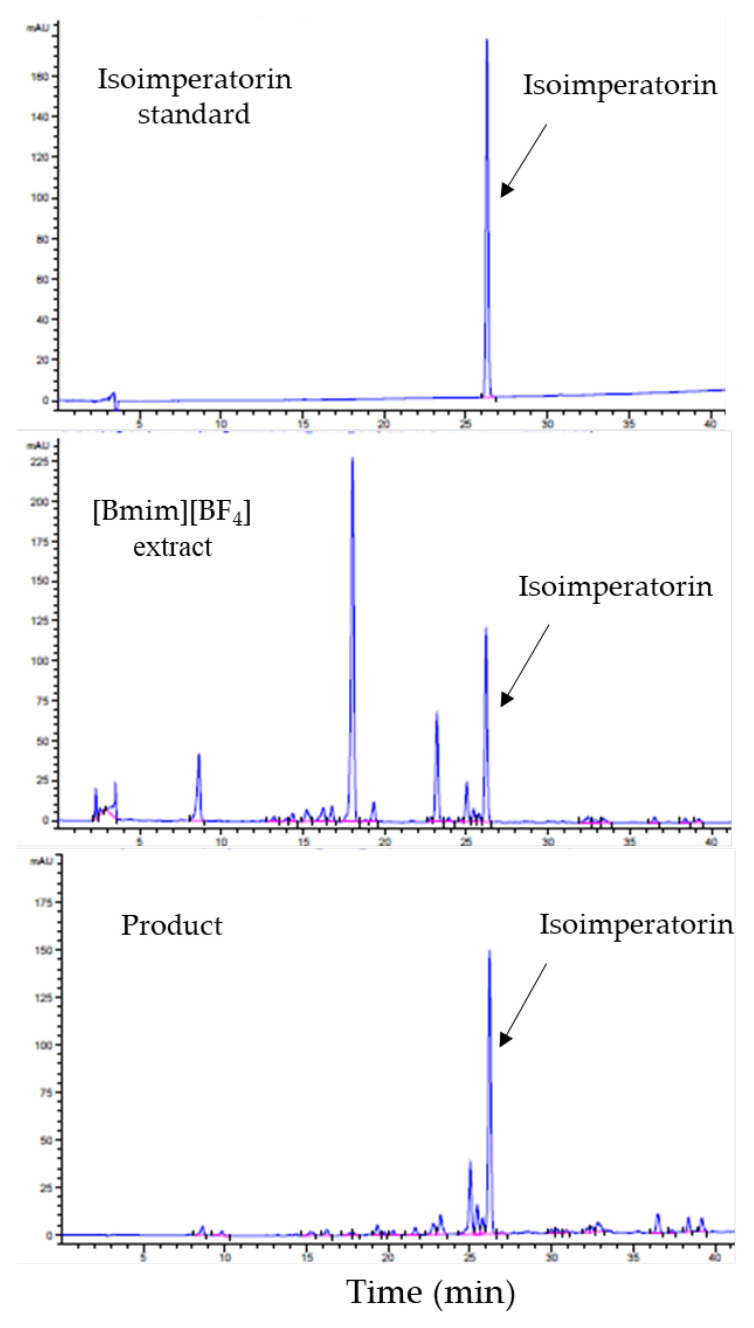
HPLC chromatographic spectra of isoimperatorin standard, the [Bmim][BF_4_] extraction solution and the product at 254 nm.

The recovery of isoimperatorin from the [Bmim][BF_4_] extraction solution was performed using a crystallization technique, namely by adding antisolvent to the extraction solution. Because preliminary crystallization studies (Section 2.1) demonstrated that water was a suitable antisolvent to precipitated isoimperatorin from the [Bmim][BF_4_] extraction solutions, several ratios (1:0.5, 1:1, 1:15, 1:2, 1:2.5 and 1:3 (*v/v*)) of water to IL solutions were evaluated in order to enhance both the recovery yield and the content (purity) of isoimperatorin in the obtained products. The initial amount of isoimperatorin was 2.58 mg. The volume of the [Bmim][BF_4_] IL extraction solution was 19 mL. The samples were allowed to precipitate at room temperature (20 °C) for 12 h. As a result, the highest recovery rate of 2.29 mg (88.76%) of isoimperatorin was reached at a 1:2.5 ratio (Table 3). The increase of the isoimperatorin saturation level in the crystallization system with the increment of water content is presumed to be the driving force leading to the enhancement of the isoimperatorin recovery yield. In this work, verification experiments were conducted at a 1:25 ratio to confirm the result. The initial amount of isoimperatorin was the same as above. As illustrated in Table 4, the rate of recovery of isoimperatorin reached 2.26 ± 0.06 mg (87.73 ± 2.37%), which was a satisfactory result and was in accordance with the result obtained in Table 3. The purity of the isoimperatorin in the final products increased up to 26.94 ± 1.26%. This improvement was nearly 103-folds greater than isoimperatorin purity in the raw material (0.26 ± 0.28%). It is also important to note that the recovery and enrichment of isoimperatorin were successfully achieved in a one-step crystallization process. The HPLC chromatographic spectrum of the final product is illustrate in Figure 5.

## 3. Materials and Methods

### 3.1. Reagents and Materials

Dried roots of *O. koreanum* were purchased from an oriental herbal medicine market in Seoul, Korea, in 2019. The plant material was identified by professor Kiwon Jung. A voucher specimen has been deposited in the herbarium of the college of pharmacy, CHA University, with registration number HPC-AR02. Isoimperatorin standard of HPLC purity > 95% was directly separated from the roots of *O. koreanum*. All ILs employed in this work, [Bmim][Br], [Bmim][Cl], [Bmim][BF_4_], [Bmim][PF_6_], and [Bmim][TFSI] were purchased from Wonsang P&C Co., Ltd. (Anyang, South Korea). Analytical and chromatography grade reagents were all purchased from Daejung Chemicals (Siheung, South Korea). Water was purified using a PURELAB^®^ chorus purification system (ELGA LabWater, High Wycombe, UK).

### 3.2. Methods

#### 3.2.1. Ionic Liquid Assisted-Extraction of Isoimperatorin

Dried roots of *O. koreanum* were ground and sieved through a 50 mesh. Powdered samples of 1.0 g each were mixed with pure, aqueous or EtOH solutions of [Bmim][Br], [Bmim][Cl], [Bmim][BF_4_], [Bmim][PF_6_], and [Bmim][TFSI] ILs in 20 mL flasks. The content of IL in the cosolvent system was fixed as one mole per liter (1.0 mol/L). The samples were then extracted on a hot plate for 30 min and sonicated at 40 kHz frequency (Branson CPX5800H Ultrasonic cleaner, Emerson Electric, Co., St. Louis, MI, USA) for 30 min. The ultrasonic power was fixed at 150 W. The extraction temperature was set at 40 °C and the rotational speed was 600 rpm. The extraction conditions were designed and optimized by the univariate method and by RSM, using central composite design (CCD) and Minitab 16 software (Minitab, LLC, State College, PA, USA). The variables were: extraction time on hot plate (30–150 min), sonication time (0–60 min), extraction temperature (20–80 °C) and solid to liquid ratio (1:5–1:30). After extraction, the samples were centrifuged at 13,000× *g* for 10 min. Thereafter, 50 μL aliquot of each sample was diluted to make 1 mL solution and then injected into HPLC for analysis. The experiments were duplicated and data were reported as mean values. The results were calculated using the following equation:

(2) $$\mathrm{Extraction}\;\mathrm{yield}\;\left(\%\right)=\frac{M_{1\;}}{M}\times 100$$

where M is the content of isoimperatorin in the plant material and M_1_ the content of the isoimperatorin in the extract.

#### 3.2.2. High-Performance Liquid Chromatography (HPLC) Analyses

HPLC analyses were conducted on an Agilent 1260 Infinity Quaternary LC system (Santa Clara, CA, USA) equipped with a G1315D UV-detector at 254 nm. The analyses were performed on an Aegispak C18-L (5 μm, 4.6 mm × 250 mm, YoungJin Biochrom, Seongnam, Korea) analytical column eluted using a gradient of 0.1% (*v/v*) phosphoric acid-water and acetonitrile from 70:30 to 10:90 over 42 min. The injection volume was set at 20 μL, the flow rate at 1.0 mL/min, and the column temperature was maintained at 30 °C. The data acquisition and analysis were conducted via Agilent OpenLab software. The calibration curve of the isoimperatorin standard was constructed by plotting the peak area ratios versus the concentrations (2.5–100 μg. mL^−1^). The regression coefficient R^2^ = 0.9997 demonstrated that the model was sufficient for the quantitative determination of isoimperatorin within the calibration range. The method showed good accuracy and precision as well. The regression equation was:

Y = 69.3160x − 46.7373(3)

#### 3.2.3. Determination of Coumarins from the Roots of *O. koreanum*

The content of isoimperatorin in the roots of *O. koreanum* was evaluated by extracting 1.0 g of the plant material with 98% EtOH (30 mL × 4) until the targeting component was undetected in the extraction solution. Later, extracts were dried under vacuum and analyzed by HPLC. The experiments were conducted in triplicate. From the experiments, the average content of isoimperatorin was found to be 2.63 ± 0.28 mg/g (0.26 ± 0.28% of raw material). The value was close to that reported by Kim, S. et al. (2011) regarding *O. koreanum* which originated from the southern region of Korea [45]. Therefore, the above value was considered as the standard amount of isoimperatorin in the roots of *O. koreanum*, and was used as a reference for comparative experiments.

#### 3.2.4. Recovery of Isoimperatorin from the [Bmim][BF_4_] IL Extraction Solution

Crystallization analyses were carried out for the purpose of recovering isoimperatorin from the [Bmim][BF_4_] extraction solution. MeOH, EtOH, and water were investigated as crystallization solvents. Precipitate was solely produced when water was employed. The obtained product was collected, vacuum dried (40 °C), and then analyzed by HPLC to determine the nature of the product as well as the content of isoimperatorin. Further, experiments were conducted at varied ratios of water to [Bmim][BF_4_] extraction solution. The aim was to maximize the rate of recovery and enhance the purity of isoimperatorin in the products. The ratios were set as follows; 1:0.5, 1:1, 1:1.5, 1:2, 1:2.5 and 1:3 (*v/v*). Following this, the samples were allowed to stand at room temperature until crystallization was completed (for 12 h). All of the obtained products were washed with water to eliminate any remaining IL, were vacuum dried (40 °C) overnight, and then analyzed through HPLC. The following equations were used to calculate the recovery and the content of isoimperatorin in the product:

(4) $$\mathrm{Recovery}\;\left(\%\right)\;=\frac{M_{1}}{M}\times 100$$

(5) $$\mathrm{Content}\;\left(\%\right)\;=\frac{M_{2}}{M_{4}}\;\times 100$$

where M and M_1_ are the same as in Equation (3), M_2_ is the content of isoimperatorin in the product and M_3_ is the total amount of the product.

#### 3.2.5. Structural Identification

The ^1^H-NMR and ^13^C NMR spectroscopic analyses of isoimperatorin standard compound extracted from *O. koreanum* were performed in CDCl_3_ solvent, on 800 MHz and 200 MHz NMR spectrometers (Advance, Bruker, Billerica, MA, USA), to assess the compound’s chemical structure. The ^1^H NMR and ^13^C NMR spectra of isoimperatorin were assigned as: ^1^H NMR (CDCl_3_, 800 MHz) δ 8.14 (dd, *J* = 0.4, 9.7 Hz, 1H), 7.57 (d, *J* = 2.3 Hz, 1H), 7.14 (s, 1H), 6.94 (dd, *J* = 2.3, 0.9 Hz, 1H), 6.25 (d, *J* = 9.7 Hz, 1H), 5.52 (tq, *J* = 7.0, 1.4 Hz, 1H), 4.90 (d, *J* = 7.0 Hz, 2H), 1.78 (s, 3H), 1.68 (s, 3H); ^13^C NMR (CDCl_3_, 200 MHz) δ 161.3, 158.1, 152.7, 148.9, 144.9, 139.8, 139.6, 119.1, 114.2, 112.6, 107.5, 105.0, 94.2, 69.7, 25.8, and 18.2, and the spectra corresponded with those reported in the literature [54,55].

## 4. Conclusions

This work successfully developed an efficient IL-based extraction and crystallization approach for the separation, recovery and enrichment of isoimperatorin. Several factors were investigated and optimized by RSM. The optimum extraction conditions were: [Bmim][BF_4_] as extraction solvent, 46 min extraction time, and 1:19 solid to liquid ratio. Under these conditions, the extraction yield of isoimperatorin increased up to 97%. In addition, crystallization, using water as an antisolvent, was successfully performed to recover isoimperatorin from the [Bmim][BF_4_] solution. The recovery yield of isoimperatorin reached approximately 88%, while its purity improved nearly to 26.9% in the product in a one-step crystallization process. An improvement greater than 100-folds was reached. Generally, conventional techniques (using organic solvents) used for the separation of phytochemicals are tedious, time-consuming and involve the use of large amounts of volatile organic solvents. To overcome these limitations, ILs have been effectively utilized. The use of ILs has a great impact on improving the selectivity and extraction efficiency of several phytochemicals as well. However, the recovery of targeting compounds from the IL solutions remains a big challenge, partially due to ILs’ non-volatile nature. Nevertheless, in the present study, not only high extraction yield of isoimperatorin was successfully achieved using IL, but also its recovery and enrichment from the IL solution were satisfactorily reached. The developed approach remarkably lessened the laboriousness of the process, reduced the time and the use of volatile organic solvents, which are drawbacks encountered in the traditional methods employed for acquiring isoimperatorin. The simplicity and effectiveness of the method are assumed to be beneficial for producing highly enriched isoimperatorin products as well as promoting its purification into a single component. The accessibility of isoimperatorin rich-product can significantly help to enhance its application for scientific research.

## Figures and Tables

**Table 1:** Experimental matrix according to the central composite design (CCD) and response for extraction of isoimperatorin.

Run	X_1_ (min)	X_2_ (g/mL)	Concentration (ug)		Concentration (%)
			Predicted	Experimental	Experimental
1	30	1:5	1.35	1.29	48.88
2	30	1:20	2.52	2.45	93.02
3	120	1:5	1.45	1.38	52.31
4	120	1:20	2.65	2.57	97.62
5	75	1:2	0.95	1.02	38.86
6	75	1:23	2.61	2.68	102.05
7	11.5	1:12.5	2.12	2.19	83.31
8	140	1:12.5	2.29	2.37	90.09
9	75	1:12.5	2.45	2.39	90.97
10	75	1:12.5	2.45	2.40	91.24
11	75	1:12.5	2.45	2.42	92.12
12	75	1:12.5	2.45	2.55	96.95
13	75	1:12.5	1.35	2.47	93.99

**Table 2:** Analysis of Variance (ANOVA).

	Degrees of Freedom	Sum of Squares	*F*-Value	*p*-Value
Model	5	5219.74	82.97	0.0001
X_1_	1	38.75	3.08	0.123
X_2_	1	3997.11	317.69	0.0001
X_1_^2^	1	146.14	11.62	0.011
X_2_^2^	1	1037.39	89.25	0.0001
X_1_X_2_	1	0.34	0.03	0.873
Residual	7	88.07	-	-
Lack of fit	3	63.46	3.44	0.132
Pure error	4	24.61	-	-
Total	12	5307.81		
R^2^		98.34%		
R^2^ -adj		97.16%		

**Table 3:** Recovery rates of isoimperatorin at different ratios of the [Bmim][BF_4_] IL extraction solution to DW.

Ratios	Recovery Yield of Isoimperatorin (mg)	Recovery Yield of Isoimperatorin (%)
1:05	0.62	24.03
1:1	1.31	50.78
1:1.5	1.76	68.22
1:2	2.04	79.07
1:2.5	2.29	86.43
1:3	2.12	82.17

**Table 4:** Verification recovery experiment and purity of isoimperatorin in the product.

Run	Weight of the Product (mg)	Recovery Yield of Isoimperatorin (mg)	Recovery Yield of Isoimperatorin (%)	Purity of Isoimperatorin (%)
1	9.12	2.33	90.31	25.55
2	8.03	2.25	87.21	28.02
3	8.11	2.21	85.66	27.25
Average	8.42	2.26	87.73	26.94
Standard deviation	0.61	0.06	2.37	1.26

## Sample Availability

Sample of the compound isoimperatorin is available from the authors.
